# Expression and subcellular localization of aquaporin water channels in the polarized hepatocyte cell line, WIF-B

**DOI:** 10.1186/1472-6793-5-13

**Published:** 2005-08-18

**Authors:** Sergio A Gradilone, Pamela S Tietz, Patrick L Splinter, Raúl A Marinelli, Nicholas F LaRusso

**Affiliations:** 1 Instituto de Fisiología Experimental, Consejo Nacional de Investigaciones Científicas y Técnicas (CONICET), Facultad de Ciencias Bioquímicas y Farmacéuticas, Universidad Nacional de Rosario, 2000 Rosario, Santa Fe, Argentina; 2 Center for Basic Research in Digestive Diseases. Mayo Medical School, Clinic and Foundation, Rochester, MN, USA

## Abstract

**Background:**

Recent data suggest that canalicular bile secretion involves selective expression and coordinated regulation of aquaporins (AQPs), a family of water channels proteins. In order to further characterize the role of AQPs in this process, an *in vitro *cell system with retained polarity and expression of AQPs and relevant solute transporters involved in bile formation is highly desirable. The WIF-B cell line is a highly differentiated and polarized rat hepatoma/human fibroblast hybrid, which forms abundant bile canalicular structures. This cell line has been reported to be a good *in vitro *model for studying hepatocyte polarity.

**Results:**

Using RT-PCR, immunoblotting and confocal immunofluorescence, we showed that WIF-B cells express the aquaporin water channels that facilitate the osmotically driven water movements in the liver, i.e. AQP8, AQP9, and AQP0; as well as the key solute transporters involved in the generation of canalicular osmotic gradients, i.e., the bile salt export pump Bsep, the organic anion transporter Mrp2 and the chloride bicarbonate exchanger AE2. The subcellular localization of the AQPs and the solute transporters in WIF-B cells was similar to that in freshly isolated rat hepatocytes and in intact liver. Immunofluorescent costaining studies showed intracellular colocalization of AQP8 and AE2, suggesting the possibility that these transporters are expressed in the same population of pericanalicular vesicles.

**Conclusion:**

The hepatocyte cell line WIF-B retains the expression and subcellular localization of aquaporin water channels as well as key solute transporters for canalicular bile secretion. Thus, these cells can work as a valuable tool for regulatory and mechanistic studies of the biology of bile formation.

## Background

Hepatocytes are polarized epithelial cells that possess well defined apical (canalicular) and basolateral (sinusoidal) plasma membrane domains. Bile secretion involves the movement of water across hepatocyte plasma membrane domains in response to transient osmotic gradients generated by active solute transport into the canalicular space of bile acids, glutathione and bicarbonate [1]. The main canalicular solute transporters are the bile salt export pump Bsep, the organic anion transporter Mrp2 and the chloride bicarbonate exchanger AE2 [2,3].

We and others recently reported that hepatocytes express aquaporin (AQP) water channels [4-10], a family of integral membrane proteins that increase cell membrane water permeability facilitating passive osmotically driven water movement [11,12]. In hepatocytes, the water channels aquaporin-8 (AQP8) and aquaporin-0 (AQP0) are primarily located within the cell in a vesicular compartment, and AQP8 redistributes to the canalicular membrane under a choleretic stimulus [5,10,13]. AQP8 it was recently described also localized in mitochondria [14]. Aquaporin-9 (AQP9) is found principally on the basolateral membrane [10]. Our previous studies suggest that water channels play an important role in the transcellular transport of water during primary bile secretion by hepatocytes [5,10,13,15,16].

Unfortunately, the bile formation process is difficult to study in vitro. In primary cultures of rat hepatocytes, downregulation of both basolateral and canalicular solute transporters occurs [17] and bile acid uptake gradually decreases and disappears after 1 to 4 days [18]. Moreover, in freshly isolated rat hepatocytes, canalicular bile acid secretion appears limited [19]. In addition, the vectorial transport of solutes is lost in many hepatoma cell lines such as HepG2 [20,21], HTC [22], and Fao [23].

The WIF-B cell line is a highly differentiated and polarized rat hepatoma/human fibroblast hybrid, which forms bile canaliculi-like structures [24]. This cell line has been shown to be a good model for studying hepatocyte polarity [25,26], protein secretion [24], bile acid transport [18] and protein transport [27]. Furthermore, WIF-B cells express the basolateral Na^+^-taurocholate cotransporter, Ntcp [28] and the canalicular conjugate export pump, Mrp2 [29].

To further explore the usefulness of the WIF-B cell line as an in vitro model for regulatory and mechanistic studies of bile secretion biology, we investigated the presence and localization of aquaporins and the principal solute transporters involved in canalicular bile formation.

## Results

### Expression of aquaporins and canalicular solute transporters in WIF-B cells by RT-PCR

**Figure 1:**
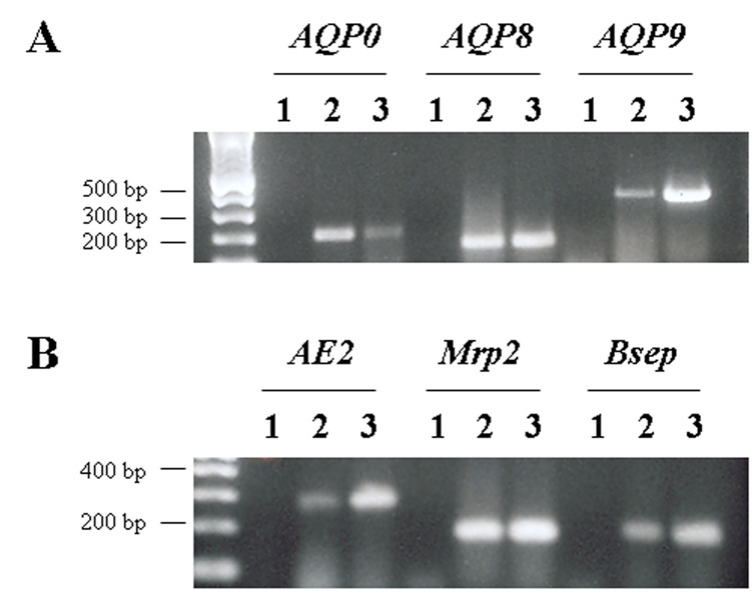
**RT-PCR for aquaporins and canalicular solute transporters in WIF-B cells**. Total RNA from WIF-B cells was reverse transcribed using random primers and then PCR-amplified with primers designed to amplify a nonconserved region of each AQP and solute transporter cDNA. Isolated rat hepatocyte cDNA were used as positive controls. *Lane *1, negative control; *lane *2, positive control; *lane *3, WIF-B cells.

RT-PCR was run for each of the three AQPs and the three solute transporters on total RNA derived from WIF-B cells. cDNA from freshly isolated rat hepatocytes was used as positive control. As shown in Figure 1, WIF-B cells express mRNA of rat AQP0, AQP8 and AQP9 (Figure 1A) and AE2, Mrp2 and Bsep (Figure 1B). The PCR products were sequenced and the identities of the amplicons, verified by data base homology searches (BLAST; NCBI, National Institutes of Health), were consistent with the predicted rat genes.

### Expression of aquaporins and canalicular solute transporters in WIF-B cells by immunoblotting

**Figure 2:**
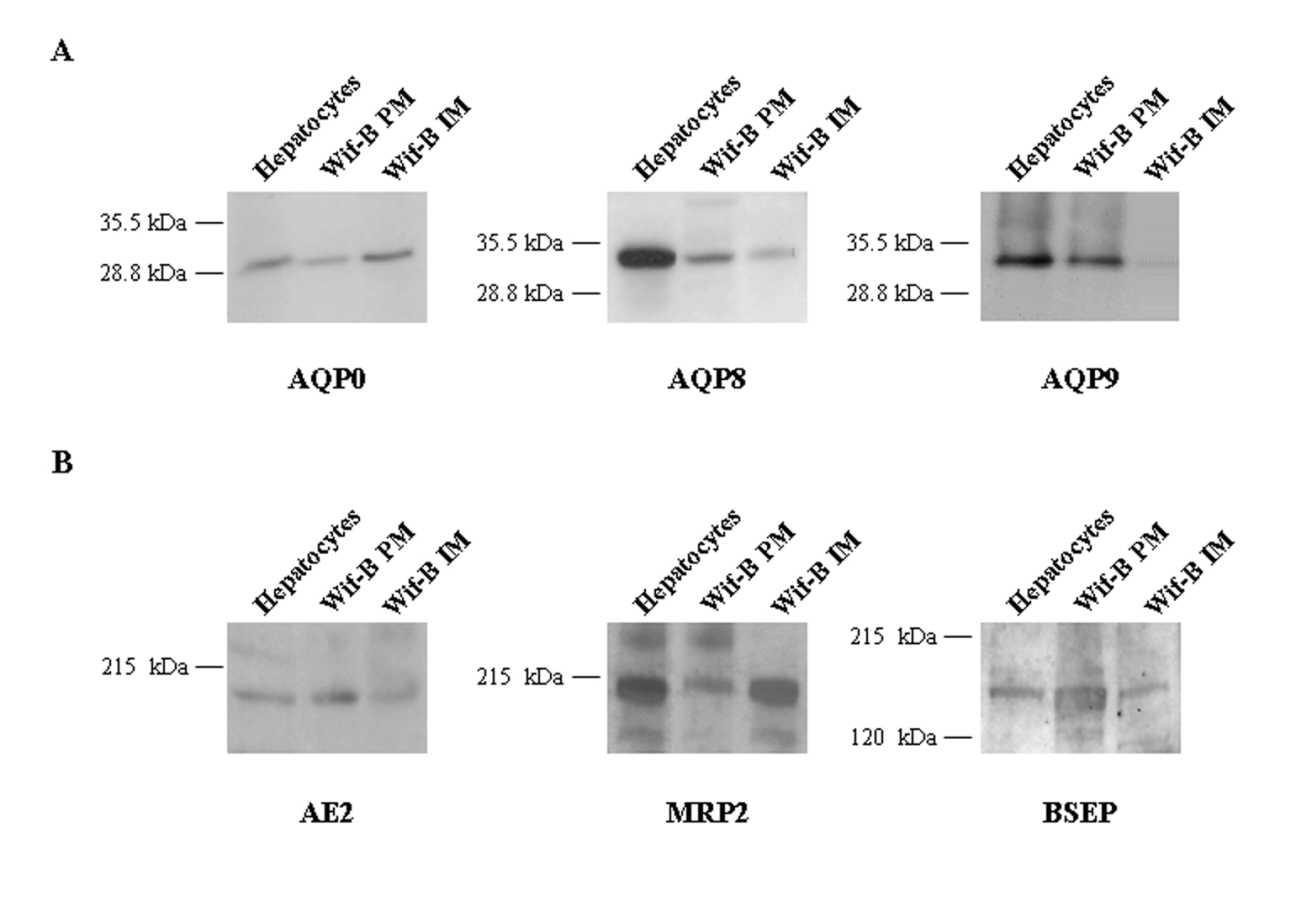
**Immunoblots for aquaporins and canalicular solute transporters in WIF-B cells**. ***A***, 50 μg of plasma (PM) and intracellular (IM) membranes from WIF-B cells were loaded onto 12% SDS-polyacrylamide gels. Immunoblots were performed using affinity-purified rabbit anti-AQP primary antibodies and goat anti-rabbit secondary antibodies and visualized via chemiluminescence. ***B***, 50 μg of PM and IM from WIF-B cells were loaded onto 8% SDS-pliacrylamide gels. Immunoblots were performed using affinity-purified goat anti-Mrp2 or Bsep and rabbit anti-AE2. Rat hepatocyte homogenate (20 μg) were used as positive controls.

As shown in Figure 2A, WIF-B cells express AQP0, AQP8, and AQP9 proteins. WIF-B plasma and intracellular membranes, as well as hepatocyte homogenates showed a 28 kDa band on the immunoblotting for AQP0. The immunoblot for AQP8 shows the presence of a 34 kDa band on WIF-B plasma and intracellular membranes. AQP9 is also positive in the plasma membrane fraction of WIF-B cells, showing the 32 kDa band. Figure 2B shows the immunoblotting for the three solute transporters: AE2 (170 kDa) and Bsep (160 kDa) are present on the plasma membrane of WIF-B and, as described [29], Mrp2 (190 kDa) is also present in the cell line.

### Confocal immunofluorescence

**Figure 3:**
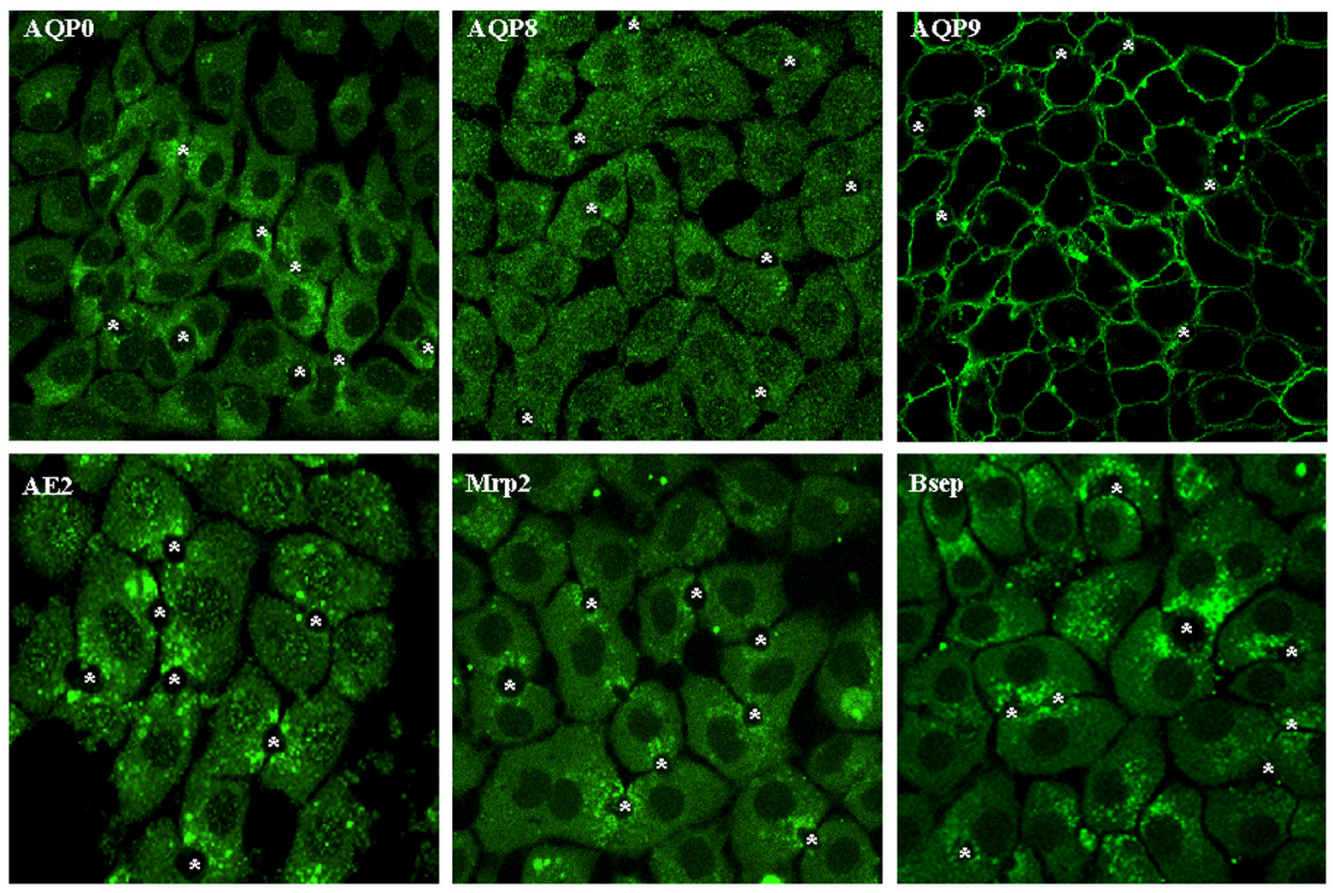
**Confocal immunofluorescence for aquaporins and solute transporters in WIF-B cells**. WIF-B cells were fixed, permeabilized, and labeled with anti-AQPs, AE2, Mrp2 or Bsep. Fluorescence localization was viewed by laser scanning confocal microscopy (see "Materials and Methods" for details). *, bile canaliculi structures.

**Figure 4:**
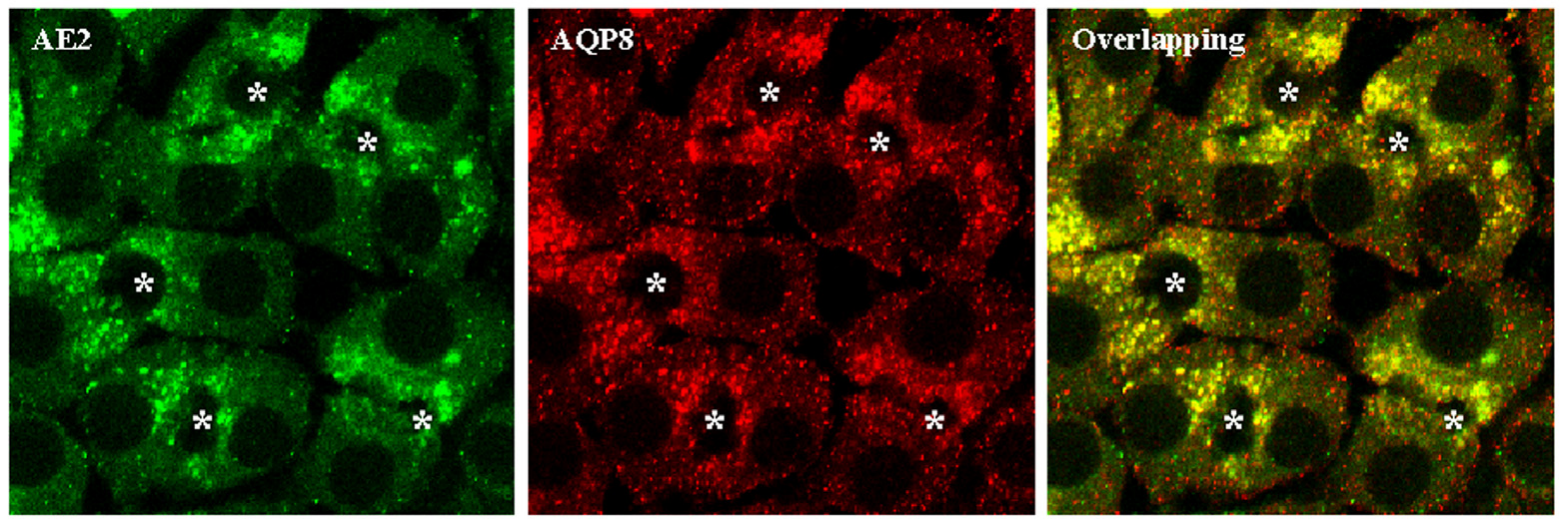
**Co-immunostaining of AQP8 and AE2 by confocal immunofluorescence**. WIF-B cells were fixed, permeabilized, and labelled simultaneously with rabbit anti-AE2 and goat anti-AQP8. Fluorescence localization was viewed by laser scanning confocal microscopy (see "Materials and Methods" for details). *, bile canaliculi structures.

Immunofluorescent staining of WIF-B cells further confirmed the expression of the three AQPs (Figure 3). The subcellular localization is similar to that of rat hepatocytes, i.e. AQP0 mainly intracellular, AQP8 mostly in intracellular vesicular structures throughout the cytosol and AQP9 on the basolateral membrane. The immunofluorescence for AE2, Bsep and Mrp2 showed pericanalicular localization (Figure 3). Immunofluorescent costaining was performed for AQP8 and AE2. These molecules showed colocalization (Figure 4), suggesting the presence of a population of pericanalicular vesicles containing both AQP8 and AE2.

## Discussion

The major finding reported here relate to the expression and subcellular localization of aquaporin water channels in the WIF-B cell line. Using RT-PCR, immunoblotting and confocal immunofluorescence we showed that (i) WIF-B cells express the aquaporin water channels that facilitate the osmotically driven water movements, i.e. AQP8, AQP9, and AQP0; as well as the key solute transporters involved in the generation of the canalicular osmotic gradients, i.e., the bile salt export pump Bsep, the organic anion transporter Mrp2 and the chloride bicarbonate exchanger AE2. (ii) The subcellular localization of the AQPs and the solute transporters in WIF-B cells was similar to that in rat isolated hepatocytes and in whole liver. (iii) Immunofluorescent costaining studies showed intracellular colocalization of AQP8 and AE2, suggesting the possibility that these transporters are expressed in the same population of pericanalicular vesicles.

Hepatocyte bile secretion is formed by passive movement of water from plasma to the bile canaliculus in response to osmotic gradients established by the active secretion of solutes. The biliary excretion of bile salts, via the bile salt transporter Bsep, glutathione, via the organic anion transporter Mrp2, and HCO_3_^-^, via the Cl^-^/HCO_3_^- ^exchanger AE2, are thought to be the major osmotic driving forces for canalicular bile flow [1]. Conceptually, the generation of bile flow is ultimately dependent on the molecular and functional expression of these transporters on the canalicular plasma membrane domain. AQPs are present in hepatocytes at both apical and basolateral plasma membrane domains as well as in intracellular vesicle compartments. Two of these AQPs can account for the water permeability of both hepatocyte plasma membrane domains, AQP8 modulating mainly the canalicular transport of water, and AQP9 facilitating its basolateral movement [5,10,13,15,16].

In order to further characterize the role of aquaporins in bile secretion, an in vitro cell system with retained polarity and expression of AQPs and the main solute transporters involved in bile formation is highly desirable. The present work shows that WIF-B cells meet these criteria, i.e. express the key solute transporters involved in the osmotic gradient generation (AE2, Bsep and Mrp2) and the channels that facilitates plasma membrane water movement (AQP8 and AQP9).

The WIF-B hybrid cell line stably retains all rat chromosomes, and only a dozen of human chromosomes [30]. The AQP8, AE2, Bsep, and Mrp2 genes have been mapped on the human chromosomes 16 [31], 7 [32], 2 [33], and 10 [34], respectively, which are not retained by the WIF line [35,36]. Furthermore, it was described that WIF-B cells express only the rat homolog of the bile salt transporter Ntcp [28], consistent with the absence of human chromosome 14, on which Ntcp has been mapped [37]. Therefore, the AQP8, AE2, Bsep, and Mrp2 expressed by these cells should be the rat genes. By contrast, the AQP0 and AQP9 genes are located on human chromosomes 12 and 15, respectively [38,39], which are present in WIF cells [35,36]. Thus, WIF-B cells may also express human AQP0 and AQP9.

The subcellular localization of the AQPs in WIF-B cells is similar to that found in isolated hepatocytes and liver from rat. AQP0 was found mainly intracellular and AQP9 exclusively on basolateral membranes. AQP8 showed mostly an intracellular vesicular structures localization, which could be of potential interest for further studies on the mechanisms involved in the hormone-regulated trafficking of AQP8 to the canalicular plasma membrane domain [5,10,13,40].

The canalicular/pericanalicular localization of the solute transporters is consistent with that described for rat liver or isolated hepatocytes. AE2 has been localized to the canalicular membrane domain [41,42]. Nevertheless, the canalicular activity of AE2 is increased in response to stimulation with cyclic AMP, and this increased activity can be blocked with colchicine, suggesting the microtubule-dependent targeting of pericanalicular vesicles containing this exchanger to the canalicular domain [43]. Rat liver Bsep is responsible for the biliary excretion of bile acids and therefore is key to the elaboration of canalicular bile [2]. Immunogold electron microscopy detection of Bsep revealed that the distribution of Bsep in rat hepatocytes is not restricted to the canalicular membrane, but also detected in vesicles close to the bile canaliculus [44]. Pericanalicular distribution of Bsep was also demonstrated by immunofluorescence staining of isolated rat hepatocyte couplets [45]. We found that Mrp2, responsible for the transport into bile of a variety of amphiphilic organic anions [2], is mainly localized on pericanalicular vesicles. This observation does not fully agree with a previous work showing an exclusively canalicular membrane localization of Mrp2 in WIF-B cells [29]. The fact that Mrp2 showed a different localization in our hands could be explained by different culture conditions. Furthermore, immunogold electron microscopy detection of Mrp2 in rat liver revealed that over 50% of Mrp2 resides in intracellular vesicles close to the canalicular membrane [46]; and pericanalicular vesicular distribution was demonstrated by confocal immunofluorescence in isolated rat hepatocyte couplets [45]. This pericanalicular localization gives rise to the possibility of studying transport trafficking to the apical membrane.

It is known that the hormone glucagon stimulates bile secretion [47,48]. As we previously described, glucagon is able to increase the osmotic water permeability of hepatocytes by triggering the translocation of AQP8 vesicles to the plasma membrane, specifically to the canalicular domain [13]. Although the actual osmotic gradients involved in glucagon-induced choleresis are unknown, these transient gradients are most likely created by the facilitated transport of HCO_3_^- ^via the canalicular Cl^-^/HCO_3_^- ^exchanger AE2 [47,48]. There is evidence to suggest that glucagon is also able to stimulate the vesicle trafficking of AE2 to the hepatocyte plasma membranes [43,48]. Since the immunofluorescence for AQP8 and AE2 showed colocalization, is attractive to speculate that the solute transporter and the water channel could be packaged in the same population of vesicles conforming a bile secretory unit as has been observed for AQP1, AE2 and the cystic fibrosis transmembrane regulator Cl^- ^channels in cholangiocytes [49]. Thus, the existence of a novel transporting organelle containing functionally related proteins, that can account for solute-driven water transport, could be proposed in hepatocytes. This organelle could contain flux proteins playing integral roles in hormone-induced bile secretion.

## Conclusion

Our study provides the first evidence of a hepatocyte cell line that retains the expression and subcellular localization of aquaporin water channels, as well as key solute transporters for canalicular bile formation, turning these cells into a valuable tool for regulatory and mechanistic studies of the bile formation biology.

## Methods

### Cell culture

WIF-B cells, kindly provided by Dr. Ann Hubbard (Johns Hopkins University, Baltimore, MD), were grown at 37°C under 5% CO_2 _in modified Ham's F12 medium supplemented with 5% fetal calf serum and 10 μmol/L hypoxanthine, 0.04 μmol/L aminopterin, and 1.6 μmol/L thymidine as described [24]. Cells were plated onto plastic dishes or glass coverslips at 3.8 × 10^4 ^cells/cm^2^. We used 10 to 14 day old cultures in all experiments, the time point at which the cells reached their maximal density and polarity [24].

### RNA isolation

Total RNA was extracted from WIF-B cells or freshly isolated rat hepatocytes using Tri-Reagent (Sigma). Cells were lysed in 1 ml of Tri-Reagent/10 × 10^6 ^cells with 5 μl of Glyco-Blue (Ambion Inc., Austin, TX) added as a co-precipitant and stored at room temperature for 5 min. After addition of 0.1 ml of 1-bromo-3-chloro-propane/1 ml of Tri-Reagent, the samples were vortexed, incubated for 15 min at room temperature, and centrifuged at 12,000 × *g *for 15 min at 4°C. The upper, aqueous phase was collected and transferred to a new tube; to this, 0.5 ml of isopropanol was added per 1 ml of Tri-Reagent used for the initial lysis. The samples were incubated for 10 min and centrifuged at 12,000 × *g *for 15 min at 4°C. After removing the supernatant, the RNA pellet was washed with 1 ml of 75% ethanol and repelleted by centrifugation at 12,000 × *g *for 15 min at 4°C. RNA was resuspended in RNA Secure solution (Ambion), and the concentration and purity were determined by spectrophotometry.

### Reverse transcription-polymerase chain reaction

5 μg of total RNA was reverse transcribed using an avian myeloblastosis virus reverse transcriptase system (Promega, Madison, WI). RNA was first incubated for 10 min at 70°C. The reaction mixture included reverse transcription buffer, 25 mM MgCl2, 10 mM deoxynucleotide triphosphates, avian myeloblastosis virus reverse transcriptase, RNasin ribonuclease inhibitor, and random primers in a final volume of 95 μl. This mixture was added to the total RNA and incubated for 10 min at room temperature and then 1 h at 42°C. Heating to 95°C for 5 min stopped the reaction. The *AQPs*, *Mrp2*, *Bsep *and *AE2 *cDNA were amplified using the polymerase chain reaction with specific primers for rat genes (Table 1). cDNA from freshly isolated rat hepatocytes and H_2_O_d _were used as positive and negative controls, respectively. The PCR products were electrophoresed in 1% agarose gels, and the bands were visualised by ethidium bromide staining. Sequencing was performed on all positive PCR products (Mayo Molecular Core Facility, Rochester, MN) to confirm the identity of the amplified genes.

### Preparation of plasma and intracellular membranes

Cells were washed and sonicated in 0.3 M sucrose containing 0.1 mM phenylmethylsulfonyl fluoride and 0.1 mM leupeptin. Plasma and intracellular membranes were obtained by differential centrifugation, as previously described [5]. Proteins in the membrane fractions were assayed according to Lowry et al. [50], using bovine serum albumin as standard.

### Immunoblotting

Solubilized membrane fractions were subjected to SDS-polyacrylamide gel electrophoresis and transferred to polyvinyldifluoride membranes. After blocking, blots were incubated overnight at 4°C with affinity-purified antibodies against AQP0, AQP8, AQP9 (1 μg/ml; Alpha Diagnostics International), AE2 (5 μg/ml; Alpha Diagnostics International), MRP2 or BSEP (5 μg/ml; Santa Cruz Biotechnology). The blots were then washed and incubated with horseradish peroxidase-conjugated goat anti immunoglobulin, and bands were detected by an enhanced chemiluminescence detection system. Autoradiographs were obtained by exposing polyvinyldifluoride membranes to Kodak XAR film.

### Immunofluorescence and confocal microscopy

After culturing, cells were fixed with 2% paraformaldehyde for 10 min at room temperature, permeabilized with 0.2 % Triton X-100 for 2 min, and incubated overnight at 4°C with affinity-purified antibodies (10 μg/ml AQP0, 10 μg/ml AQP8, 10 μg/ml AQP9, 10 μg/ml AE2, 20 μg/ml Mrp2 or 20 μg/ml Bsep). After washing, coverslips were incubated with Alexa Fluor 488 or 594 conjugated secondary antibodies for one hour, and mounted with ProLong. Fluorescence localization was detected by confocal microscopy with a laser scanning microscope (Carl Zeiss LSM-510). Images were obtained with the same confocal settings for each set of experiments. With these settings no autofluorescence was detected. Controls using omission of primary or secondary antibodies revealed no labeling. Images were processed using Adobe Photoshop software.

## Authors' contributions

SAG carried out preparation of intracellular and plasma membranes, the immunoblots and the confocal immunofluorescence studies and participated in the RT-PCR and sequence alignment and drafted the manuscript. PST participated in the western blotting and confocal immunofluorescence studies and helped to draft the manuscript. PLS carried out the cell culture and the RT-PCR studies and the sequence alignment. RAM participated in the design of the study and in the draft of the manuscript. NFL conceived of the study, and participated in its design and coordination and helped to draft the manuscript. All authors read and approved the final manuscript.

## Figures and Tables

**Table 1:** Primers used for AQPs and solute transporters expression

**Gene**	**Primers (forward; reverse)**	**Amplicon size, bp**	**cDNA sequence location**
AQP0	5'-acggctcaagagtgtttctga-3'	189	669-689
	5'-tccccacagtctctttcttcat-3'		857-836
AQP8	5'-aagaccatgctgctaattcc-3'	275	423-442
	5'-tccacaatgacagagaaacc-3'		697-678
AQP9	5'-tgttgtcattagcctcctgatc-3'	356	736-757
	5'-tgaagaaagaactggatgaacg-3'		1091-1070
MRP2	5'-ctggttggaaacttggtcgt-3'	172	3719-3739
	5'-caactgccacaatgttggtc-3'		3890-3870
BSEP	5'-cactggccttctggtatggt-3'	225	1275-1294
	5'-gcttgtagccgtctcctgac-3'		1499-1479
AE2	5'-tctcgttctgcaagagcaacc-3'	270	2527-2547
	5'-ttgttactgctgctgtctgcc-3'		2797-2777
